# Infant skull fractures align with the direction of bone mineralization

**DOI:** 10.1007/s10237-024-01902-x

**Published:** 2024-11-25

**Authors:** Siyuan Chen, Svein Kleiven, Xiaogai Li

**Affiliations:** Division of Neuronic Engineering, KTH Royal Institute of Technology, Stockholm, Sweden

**Keywords:** Abusive head trauma, Infant skull fracture, Infant finite element model, Progressive unidirectional fabric composite damage model

## Abstract

The geometry and mechanical properties of infant skull bones differ significantly from those of adults. Over the past decades, debates surrounding whether fractures in infants come from deliberate abuse or accidents have generated significant impacts in both legal and societal contexts. However, the etiology of infant skull fractures remains unclear, which motivates this study with two main components of work. Firstly, we present and implement a progressive unidirectional fabric composite damage model for infant cranial vaults to represent ductile and anisotropic properties—two typical mechanical characteristics of infant skulls. Secondly, we hypothesize that these intrinsic material properties cause injuries perpendicular to the fiber direction to dominate infant skull fractures, resulting in fracture lines that align with the direction of mineralization in the infant skull. The material model and the finite element (FE) model were verified hierarchically, and this hypothesis was verified by reconstructing two legal cases with known fall heights and implementing the above damage model into CT-based subject-specific infant FE head models. We discovered that the infant skull is more susceptible to injuries within planes perpendicular to the mineralization direction because of the anisotropic mechanical property caused by the direction of mineralization, leading to infant skull fractures aligning with the mineralization direction. Our findings corroborated the several previously reported observations of fractures on cranial vaults, demonstrating that these fractures were closely associated with sutures and oriented along the mineralization direction, and revealed the underlying mechanisms of infant skull fracture pattern. The modeling methods and results of this study will serve as an anchor point for more rigorous investigations of infant skull fractures, ultimately aiming to provide convincing biomechanical evidence to aid forensic diagnoses of abusive head trauma.

**Supplementary Information:**

The online version contains supplementary material available at 10.1007/s10237-024-01902-x.

## Introduction

Pediatric skull fractures can arise from significant forces resulting from head impacts caused by either abuse or accidents (Leventhal et al. 1993). The statistics on head injuries among infants and toddlers speak for themselves and call for more precise assessment of injury causation to separate accidents from abusive trauma. It has been claimed that infants suffering from abusive head trauma (AHT) exhibit a mortality rate approximately five times greater than those with accidental head injuries (Hinds et al. 2015). Studies from other Western countries have reported an incidence of AHT ranging from 20 to 40 cases per 100,000 annually, with a mortality rate of 20–30% (Barlow and Minns 2000; Talvik et al. 2006; Jayawant et al. 1998). However, the diagnosis of AHT remains controversial due to the lack of solid evidence definitively linking the injuries to abuse (Högberg et al. 2019). A national study from Sweden, in contrast, reported an AHT mortality rate at least ten times lower than those in other Western countries, raising the possibility of misclassification in some cases that were presumed to be related to abuse (Andersson and Thiblin 2018). On the other hand, distinguishing between abusive and accidental trauma remains an extremely challenging task, as both scenarios can result in similar skull fracture patterns. The complexity is further enhanced by the incomplete understanding of the etiology of infant skull fractures and their patterns. Identifying whether injuries are accidental or the result of abuse requires the concerted efforts of forensic experts, radiologists, pediatricians, and biomechanics researchers.

The skull consists of the cranial vault and skull base with different anatomy and ossification centers (Kleinman et al. 2015). Infant cranial vaults differ from those of adults in several distinct ways. Structurally, they consist of a single, uniform, yet porous layer instead of the structure of two layers of compact bone separated by porous cancellous bone in adults (Currey 2012). Moreover, infant skull bone, in contrast to adult skull bone, is anisotropic, and the Young’s modulus along the fiber is 10–40 times stiffer than the Young’s modulus in the direction perpendicular to the fiber (Wood 1971; Coats and Margulies 2006; Metcalf et al. 2021; McPherson and Kriewall 1980). Geometrically, infant cranial vaults have not been fully closed due to the presence of unfused soft fontanelle and sutures. Besides that, the bony development of skull bones initiates at the location of the ossification center (OC), along the mineralized collagen fibers, which are also referred to as grain fibers in some literatures (Coats and Margulies 2006; Li et al. 2017; Kriewall 1982). The mineralization of cranial bones during the entire infancy stage contributes to the age-dependent anisotropic stiffness and strength, characterized by the age dependent anisotropic ratio for infant skull bones that diminish with age (Li et al. 2017).

Owing to ethical considerations, there is a paucity of infant drop experiments using postmortem human specimens (PMHS). Weber’s studies (Weber 1984, 1985) involved dropping 50 infant cadavers from a height of 82 cm onto various surfaces to investigate skull fracture patterns. As the first systematic experimental research on infant skull fractures using the largest sample of infant PMHS, it holds great value for both infant skull biomechanics researchers and forensic investigators. However, these studies lack quantitative information on both skull fracture pattern and head impact locations. On the other hand, Prange (Prange et al. 2004) conducted compression tests and drop tests on three infant heads from 15 and 30 cm heights. In addition, more recent studies by Loyd (Loyd 2011) investigated various fall tests at different heights using the pediatric, adult, and anthropometry test device (ATD) heads. The drop tests were divided into two groups based on the drop height: ’non-destructive’ and ’destructive’. Loyd’s tests are significantly informative, providing precise data on impact location, drop height, and quantitative measurements of the resulting head impact responses, though there is a potential limitation in the reuse of experimental head samples.

Medical images such as computed tomography (CT) gives the detailed skull anatomy combined with sutures and the location of OC, therefore can be based to generate subject-specific FE head models, which have been increasingly used to study various head injuries and brain traumas. However, limited studies focused on skull fracture modeling, especially on infant skull fracture. Hajiaghamemar et al. (Hajiaghamemar et al. 2019) identified different biomechanical metrics and offered valuable threshold values associated with infant skull fractures based on 11 real-world fall reconstruction. He et al. (He et al. 2020) adopted an adaptive-remeshing framework to predict infant skull fractures through a solid mechanics perspective, but the FE model in their study only encompasses the skull and sutures, excluding the brain and other soft tissues (He et al. 2020; Coats et al. 2007). Li et al. (Li et al. 2019) reconstructed two suspected abuse cases with infant skull fractures. However, the orientation of fiber was represented as a straight line from the OC without considering the influence of the curvature of the skull, and the material models for infant skull bones used in these recent studies have not been fully validated. Ruiz et al. (Ruiz-Maldonado et al. 2023) conducted a comprehensive study on the infant skull fracture patterns from falls from low heights across various ages based on CT images of 231 subjects, offering valuable references for the study of infant skull fractures. Previous studies have primarily focused on the patterns and modeling of infant skull fractures. However, to the best knowledge of the authors, investigations into the mechanisms of infant skull fractures have not been conducted due to the intricate nature of infant skull modeling.

This work proposes a novel modeling approach that faithfully captures the mechanical properties of the infant skulls, aiming to investigate the relationship between the mechanism and patterns of infant skull fracture and the mineralization direction of skull bone. The presented material model of infant skull bone and the whole infant head model are validated hierarchically against different tests. Subsequently, the infant skull fractures of two legal cases in Swedish courts are reconstructed to investigate the etiology of infant skull fractures. This study enhances our biomechanial understanding of infant skull fracture patterns and mechanism, hopefully providing a more robust reference for forensic investigations in the assessment of contentious fractures related to issues of abuse and accidents.

## Method

### Subject-specific head model generation

**Fig. 1:**
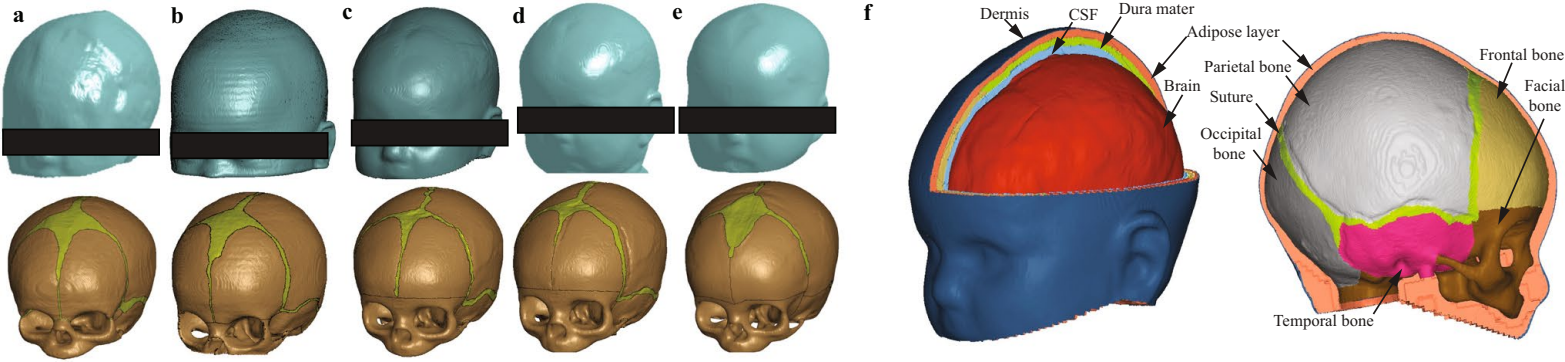
Generated infant FE head model of various age. **a** Newborn. **b** 3-month. **c** 4-month. **d** 5-month. **e** 9-month. **f** Components of the detailed 4-month FE head model

The subject-specific FE head models employed in this study were developed by Li (Li et al. 2017, 2019) based on the geometrical reconstruction of CT images, which consist of the dermis, adipose layer of the scalp, detailed infant skull bones, facial bone, sutures, dura mater, cerebrospinal fluid (CSF), and brain (Fig. 1). The total number of elements in these head models is between 3 and 6 million, and the typical size of the skull element is approximately 0.4 mm.

Since previous studies have demonstrated the importance of nonlinear biomaterial models (Li et al. 2017; Sahandifar and Kleiven 2021), the same nonlinear modeling strategy for soft tissue is employed in this study (Li et al. 2017). The infant scalp is modeled with two layers (Fahlstedt et al. 2015). The suture is modeled by the 1st-order Ogden hyperelastic model to account for the nonlinear elasticity during large deformation (Coats and Margulies 2006). The dura mater is modeled using Mooney-Rivlin hyperelastic model, with parameters determined by experimental testing of fetal dura mater (Bylski et al. 1986). A hyper-viscoelastic material model is used for brain, and incompressible fluid material model is used for CSF (Kleiven 2007). The properties of the soft materials in this study are summarized in Table 1. All simulations are carried out using LS-Dyna 13.0 with an explicit dynamic solution method.

**Table 1:** Summary of soft material properties and fitted material properties of parietal and occipital bones in different ages

Soft tissue	Material model	Material parameters	References							
*Summary of soft material properties*										
Scalp outer (Dermis)	Ogden model	$\mu _1 = 1.30 \times 10^4$ Pa, $\alpha _1 = 24.2$	Fahlstedt et al. (2015)							
Scalp inner (Adipose)	Ogden model	$\mu _1 = 3.99 \times 10^3$ Pa, $\alpha _1 = 8.8$	Fahlstedt et al. (2015)							
Suture	Ogden model	$\mu _1 = 1.48 \times 10^4$ Pa, $\alpha _1 = 6.9$	Li et al. (2017)							
Dura mater	Mooney-Rivlin hyperelastic	$C_1$ = 1.18 MPa, $C_2$ = 0.295 MPa	Bylski et al. (1986)							
Brain	Hyper-viscoelastic model	$\mu _1 = 53.8$Pa, $\alpha _1 = 10.1$, $\mu _2 = -120.4$Pa, $\alpha _2 = -12.9$	Kleiven (2007)							
CSF	Incompressible fluid model	*K* = 2.1 GPa	Kleiven (2007)							

Material properties	Newborn		3MO		4MO		5MO		9MO	
	Parietal	Occipital	Parietal	Occipital	Parietal	Occipital	Parietal	Occipital	Parietal	Occipital
*Summary of fitted material properties of parietal and occipital bones in different ages*										
$E_a$ (MPa)	4418.0	2776.3	4864.9	4310.9	5006.6	4822.4	5144.7	5333.9	5662.0	7380.0
$E_b$ (MPa)	344.3	255.9	466.0	343.3	499.0	368.9	528.6	392.9	615.9	474.6
$\sigma _a$ (MPa)	103.5	72.7	109.2	89.1	111.0	94.5	112.8	99.9	119.5	121.7
$\sigma _b$ (MPa)	23.5	12.6	30.5	15.1	32.4	15.8	34.1	16.5	39.1	18.9

### Skull bone modeling

#### Anisotropic modeling implementation

The cranial vault develops along the fiber orientation radiating from the ossification center (OC), aligning with the future eminences as early as the 7th to 8th week post-conception (PC) (Jin et al. 2016). Following the same method in previous work (Li et al. 2019), the location of the OC in various cranial bones is determined by identifying the point of maximum Gaussian curvature on the reconstructed triangular skull surfaces using the angle deficit method.

The three directions in material coordinate system of infant skull are exclusively determined by the element location, the OC position, and the normal vector of the skull outer surface. Throughout the paper, we denote variables and different material directions using italic lowercase letters, and vectors of different directions using vector notation. The vector $\overrightarrow{u}$ is defined from the OC to the center of element (CoE). The projection of vector $\overrightarrow{u}$ in the corresponding normal vector $\overrightarrow{v}$ is $\frac{\overrightarrow{u}\cdot \overrightarrow{v}}{\left| v \right| }\cdot \frac{\overrightarrow{v}}{\left| v \right| }$, where the corresponding normal vector $\overrightarrow{v}$ is determined using the K-nearest neighbors (KNN) algorithm (Peterson 2009). Then the vector of $\textit{a}$ axis can be determined as follows.

1 $$\begin{aligned} \vec {a}= \vec {u}-\frac{\vec {u}\cdot \vec {v}}{\left| v \right| }\cdot \frac{\vec {v}}{\left| v \right| } \end{aligned}$$

Considering the vector of $\textit{c}$ axis is the same as normal vector of the element along thickness direction, then the vector of $\textit{b}$ direction can be calculated based on $\overrightarrow{a}$ and $\overrightarrow{c}$.

2 $$\begin{aligned} \vec {b} = \vec {c}\times \vec {a} \end{aligned}$$

**Fig. 2:**
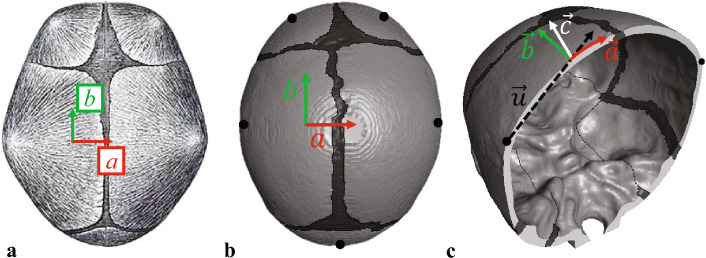
Infant skull bone anisotropic modeling. **a** Illustration of the grain fiber orientation in infant skull bone, radiating from the ossification centers of each bone platen (Gray 1918). **b** The diagram of infant skull with element exclusive material direction, the ossification centers are marked as black points. **c** The coronal section cross the ossification centers in parietal bones, and the red arrow shows the modeled fiber direction in simulation

The exclusive element material system modeling method is employed on all the skull plate bones, providing each element with three independent orthogonal directions: along the growing direction of grain fibers, the in-plane perpendicular direction, and the out-of-plane direction through the thickness (Fig. 2).

**Fig. 3:**
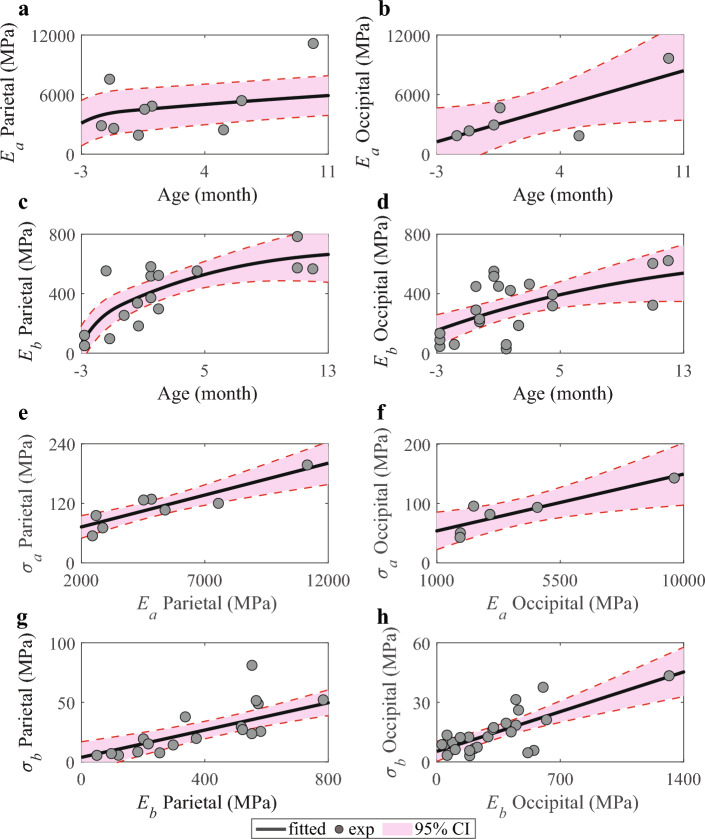
Infant skull bone anisotropic modeling. **a**–**d** The material model for $E_a$ and $E_b$ of parietal bone and occipital bone with 95% confidence interval (CI). **e**–**f** The linear fitted curve between ultimate stress and the young’s modulus of parietal bone and occipital bone along the grain fiber direction. **g**–**h** The linear fitted curve between ultimate stress and the young’s modulus of parietal bone and occipital bone along the in-plane direction

#### Material parameters

A recent study on the high-strain rate material properties of infant cranial bone indicates that Young’s modulus along the fiber direction is approximately ten times greater than that perpendicular to the growing direction of grain fibers and that a stiffer bone generally has higher ultimate stress (Metcalf et al. 2021). The Young’s modulus in different directions of the parietal bones and occipital bones are fitted as a function of age by a piecewise spline function based on the high-rate three-point-bending test results (Coats and Margulies 2006; Metcalf et al. 2021). The fitted curves are constrained to be monotonically increasing and concave down to accord with the biological growth rule (Savageau 1980), and the relationship between ultimate stress and Young’s modulus is fitted as a linear function, where the ultimate stress is calculated based on Timoshenko’s beam theory (Coats and Margulies 2006; Metcalf et al. 2021). The properties of the parietal and occipital bones developed in this study are summarized in Table 1. The relationship between yield stress and ultimate stress is assumed to be as shown below, where the factor *k* is calibrated through the 3-point bending simulation using the progressive damage model,

3 $$\begin{aligned} \sigma _y=k\cdot \sigma _u \end{aligned}$$

where $\sigma _y$ is the yield stress and $\sigma _u$ is the ultimate stress.

Given that human bones are stronger in compression than in tension (Simkin and Robin 1973; Cezayirlioglu et al. 1985; Reilly et al. 1974; Reilly and Burstein 1975; Mirzaali et al. 2016), particularly along the transverse direction (Reilly and Burstein 1975), the relationship between ultimate compressive strength and ultimate tensile strength is assumed to be identical to that of the femur bone, which is a typical anisotropic bone in humans (Reilly and Burstein 1975).

4 $$\begin{aligned} &  \sigma _{a,uC}=1.5\cdot \sigma _{a,uT} \end{aligned}$$

5 $$\begin{aligned} &  \quad \sigma _{b,uC}=2.5\cdot \sigma _{b,uT} \end{aligned}$$

The transverse isotropic material has five independent material constants (Milton and Sawicki 2003), the constraint between $G_{bc}$ and $\mu _{bc}$, $E_b$ and the constraint between $\mu _{ab}$, $E_a$ and $\mu _{ba}$, $E_2$ are shown below.

6 $$\begin{aligned} &  G_{bc}=\frac{E_b}{2(1+\mu _{bc})} \end{aligned}$$

7 $$\begin{aligned} &  \quad \frac{\mu _{ab}}{E_a}=\frac{\mu _{ba}}{E_b} \end{aligned}$$

Considering Eqs. (6, 7), the $G_{ab}$ is approximated as follows.

8 $$\begin{aligned} G_{ab}=\frac{\sqrt{E_aE_b}}{2(1+\sqrt{\mu _{ab}\mu _{ba}})} \end{aligned}$$

Based on the phenomenon that the shear yield strain is similar to tensile yield strain for healthy bone (Mirzaali et al. 2016), the shear yield stress can be estimated as follows.

9 $$\begin{aligned} &  S_{ab,y}=G_{ab}\cdot \varepsilon _{ab,y} \approx G_{ab}\cdot \varepsilon _{b,y}=G_{ab}\cdot \frac{\sigma _{b,y}}{E_b} \end{aligned}$$

10 $$\begin{aligned} &  \quad S_{bc,y}=G_{bc}\cdot \varepsilon _{bc,y} \approx G_{bc}\cdot \varepsilon _{b,y}=G_{bc}\cdot \frac{\sigma _{b,y}}{E_b} \end{aligned}$$

#### Anisotropic constitutive model with progressive damage

The infant skull differs significantly from the adult skull in ductile properties in different directions (Coats and Margulies 2006; Metcalf et al. 2021), while the typical failure material model cannot reflect the strain-softening behavior of the infant skull under mechanical testing. In this study, the progressive damaged model based on the three-dimensional failure theory of unidirectional fabric composite (Hashin 1980; LST 2002) was used to represent the material behavior of infant skull bones. The detailed modeling and formulation of the employed progressive damaged model in this study can be found in the Appendix A.

### Hierarchical validation against experimental data

#### Three-point bending test

Coats et al. (Coats and Margulies 2006) conducted tests on the material properties of the infant skull along the perpendicular fiber direction at high rates. To validate the material model and calibrate the damage parameters, the specimen number 17 was selected for validation because of the availability of detailed property information and the complete force-displacement curve. Metcalf et al. (Metcalf et al. 2021) conducted high-rate three-point bending tests on infant cranium specimens with the long axis aligned with the fiber direction. In total 6 tests corresponding to the distinguished strain stress curve are simulated with the same damage parameters.

#### Validation of infant head model for low-height drop test

The global performance of the whole-head infant FE model is evaluated by comparison with Loyd’s low-height drop tests at the same impact angle at the same height (futher details can be found in Chapter 6 of Loyd (2011)), which involve three specimens of different ages: −1.35M (refer as P13F in test), 5 M (P12M), and 9 M (P14M). Since acceleration-time curves for P12M were not available, a comparison was made with the test using maximum acceleration and impact stiffness. The impact stiffness was determined by analyzing the linear segment of the force-displacement curve, following the same method employed in the test. Impact velocities of 1.71 and 2.42 m/s were assigned to low-height drop simulations to represent the free-drop tests from heights of 15 cm and 30 cm, respectively.

To account for the impact of individual variations of Young’s modulus within the infant skull on the results, in addition to the fitted material model shown in Sect. 2.2.2, each case is also simulated using the lower and upper values of the 95% CI boundary to represent the compliant and stiff skull scenarios. CORA (CORrelation and Analysis, version 3.6.1) scores are used to evaluate the correlation between experimental and simulated results, employing the cross-correlation method described in CORA. Following the methodology reported by Li et al. (2021), the CORA score in this study is computed as the average of the shape (V), size (G), and phase (P) scores: $((V+G+P)/3$. CORA scores range from 0 to 1, where 1 represents a perfect correlation.

#### Infant skull fracture: validation with Weber’s experiments

Weber (Weber 1984, 1985) investigated the fracture patterns of infant skulls impacted on the parietal-occipital bone. Among the 15 cases in which the head was dropped onto a hard impact surface, two cases of different ages and distinct fracture patterns were chosen to validate the skull fracture reconstruction capability of the model. Case A2 displays a pattern of multiple fractures in the occipital bone of a 4-month-old infant, whereas case C5 demonstrates a pattern of a single linear fracture in the right parietal bone of a newborn. The impact velocity is set to match the free-fall velocity from the experimental drop height, and the material model is based on the fitted model described in Sect. 2.2.2.

### Two legal cases with skull fracture

Two legal cases of non-abused 3- and 4-month-old infants are reconstructed using their respective subject-specific FE head model, followed by a comparison with the actual skull fracture. Both cases have been authorized to be used in the present investigation, and comprehensive details about these two cases can be found in a prior study (Li et al. 2019). The 3-month-old baby experienced a fall from an estimated height of 0.84 m, while the 4-month-old infant fell from an estimated height of 1.1 m. The potential impact locations were determined based on the maximum point of swelling. The initial velocity in the simulation is approximated as the free fall velocity from the corresponding height. Each case is simulated using three different groups of material parameters to assess the impact of individual variations in skull mechanical characteristics on the fracture pattern. Apart from the general group represented by the fitted material property line, the Young’s modulus of the 95% CI lower boundary, in conjunction with the 95% CI lower strength values and the Young’s modulus of the 95% CI upper boundary, along with the 95% CI higher strength values in Fig. 3 are used to comparison. Furthermore, the fracture pattern in three directions is evaluated and compared to validate the hypothesis.

## Result

### Model validation against multiple experiments

#### Three-point bending test simulation

**Fig. 4:**
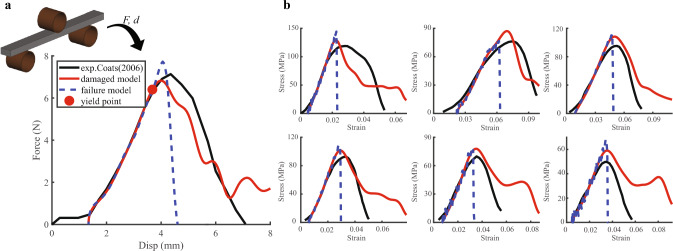
The three-point bending test results. **a** One case from Coats et al. (Coats and Margulies 2006) with the long axis along the fiber direction. Both the failure model (represented by the blue dashed line) and the damage model (in red) curves were filtered using the ’SAE 600 sec’ setting in LS-Prepost 4.8 to smooth out fluctuations. The linear stages are identical before the yield point (marked with a red point). **b** Six cases from Metcalf et al. (Metcalf et al. 2021) with the long axis perpendicular to the fiber direction, corresponding from top left to bottom right to subjects 6, 7, 8, 3, and parietal bone and occipital bone of subject 1 in their tests (Metcalf et al. 2021). No filter was applied to the failure model curve (in blue) to better emphasize that the failure model does not exhibit ductile properties

The simulated force-displacement curve of the test by Coats et al. (Coats and Margulies 2006) is shown in Fig. 4a. The simulated result from the damage model used in this study is also compared with the result of previous failure model.

Contrast to the failure material model without damage, the progressive damage model can better represent the ductile properties of the infant skull. In the initial linear stage, the slope of the simulated curve is consistent with the test curve until the yield point (marked in red), where the maximum stress of the specimen reaches the yield stress for the first time. During the initial stage of damage propagation, the curve continues to ascend with a decreasing slope until the force reaches its maximum.

#### Low-height drop simulation

**Fig. 5:**
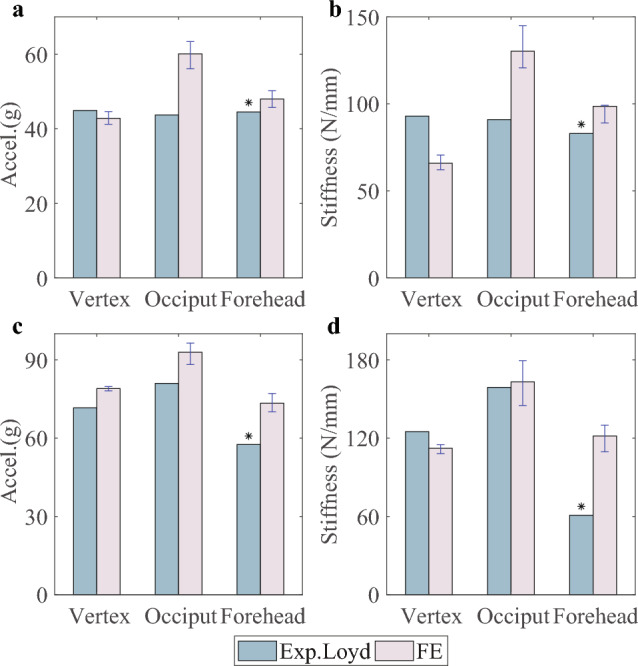
Simulated and experimental maximum acceleration and stiffness for the impacts at three different locations for the 5 M case (P12M). * indicates the special case where the peak acceleration is higher under the 15 cm than 30 cm. **a** The maximum acceleration at a drop height of 15 cm. **b** The stiffness at a drop height of 15 cm. **c** The maximum acceleration at a drop height of 30 cm. **d** The stiffness at a drop height of 30 cm

**Fig. 6:**
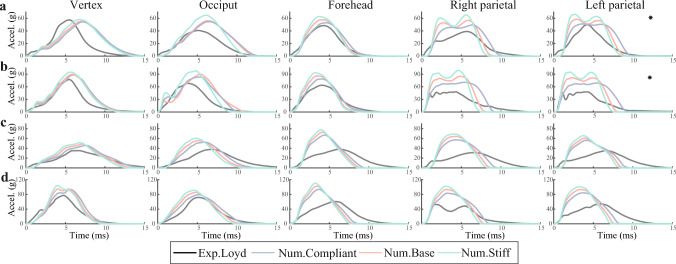
Simulated and experimental acceleration time curves for the impacts at five different locations for the newborn (P13F) and the 9 M specimen (P14M). * means the special case where the peak acceleration under the 15 cm lowers than 30 cm. For each drop scenario, simulations were performed with three different sets of skull material parameters: the ’compliant’ group used the elastic modulus at the lower bound of the 95% CI, the ’base’ group used the fitted elastic modulus of the skull, and the ’stiff’ group used the elastic modulus at the upper bound of the 95% CI, as described in Sect. 2.2.2. **a** P13F at a drop height of 15 cm. **b** P13F at a drop height of 30 cm. **c** P14M at a drop height of 15 cm. **d** P14M at a drop height of 30 cm

The comparisons between the numerical results with different material parameters and the actual low-height drop test results are shown in Figs. 5 and 6. For P12M, the maximum acceleration and stiffness during the impact are calculated using the same method with the test ( Loyd (2011), Chapter 6), and the variations between the compliant and the stiff group are plotted. Among the three impact locations, the occiput impact results in the highest acceleration and stiffness. For P13F and P14M, the acceleration-time impact curves are compared with the test curves, where the black curve represents the test result, the red curve is the simulated result using cranial bone material parameters derived from the fitted Young’s modulus curve in Sect. 2.2.2, and the blue and green curves are the simulated results using cranial bone material parameters determined by the 95% CI boundary curves. The impact position significantly influences the outcomes across different scenarios. The final average CORA scores for the acceleration-time impact curves are presented in Table 2. For most impact scenarios, the compliant material group exhibits the best correlation with the corresponding test curve, except for the P13F 15 cm impact on the Vertex. For P13F, all CORA scores in the compliant group exceed 0.7, which can be rated as "good" from the ISO/TR-9770 (ISO 2002), as used previously (Giordano and Kleiven 2016). However, the performance of P14M is not as good, especially for the forehead, right parietal, and left parietal impacts at 15 cm, and forehead impact at 30 cm, with all scoring below 0.5. The low CORA scores for these impact scenarios primarily result from the low phase (*P*) scores, which may be caused by discrepancies between the actual and simulated subjects.

**Table 2:** Average CORA scores for the acceleration-time impact curves of the low-height drop validation (P13F and P14M)

	P13F 15 cm			P14M 15 cm		
Impact location	Compliant	Base	Stiff	Compliant	Base	Stiff
Vertex	0.803	0.857	0.87	0.869	0.849	0.819
Occiput	0.791	0.789	0.778	0.493	0.478	0.435
Forehead	0.912	0.889	0.86	0.4	0.365	0.337
Right parietal	0.813	0.778	0.616	0.367	0.313	0.277
Left parietal	0.81	0.775	0.75	0.398	0.359	0.317
	P13F 30 cm			P14M 30 cm		
Vertex	0.883	0.875	0.85	0.825	0.821	0.819
Occiput	0.813	0.777	0.792	0.901	0.842	0.795
Forehead	0.896	0.709	0.68	0.484	0.43	0.39
Right parietal	0.741	0.738	0.709	0.745	0.714	0.683
Left parietal	0.718	0.715	0.701	0.684	0.594	0.558

#### Fracture comparison with Weber’s experiments

**Fig. 7:**
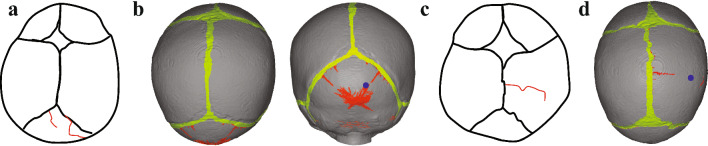
The comparison of the simulation results and experiment result for Weber’s cases. The suture is plotted in green, the damage elements are in red and the impact point is represent as blue dot. **a** Weber’s record of case A2 (4 M). **b** The simulation result of case A2. **c** Weber’s record of C5 (Newborn). **d** The simulation result of case C5

The simulation results of Weber’s cases are shown in Fig. 7 with the reconstructed fractures highlighted in red. In the case of A2, the simulation results identify four significant fractures within the occipital bone, with the primary fracture beginning near the ossification center (OC) and extending parallel to the right lambdoidal suture. In particular, the fracture pattern in the A2 simulation reveals a localized flaky damage region near the OC. Additionally, the simulation results indicate two fractures on the left side; one long and one short, both originating from positions near the same suture and extending toward the OC. In Weber’s experiments, only two linear fractures were recorded: a shorter one on the left, extending from the left lambdoidal suture toward the OC, and another on the right, close to the lambdoidal suture. Both fractures are reconstructed in our FE simulation. Notably, the right fracture in Weber’s tests initially diverges toward the OC from the right lambdoidal suture before running parallel to it, matching closely with our simulation results.

For the case of C5, the fracture predicted by the simulation initiates at the midpoint of the sagittal suture and then extends toward the right parietal bone in an approximately perpendicular direction to the suture. Similarly, the end of the fracture observed by Weber’s experiment also started at the same position and developed in the same general direction toward the OC. However, the head shape of Weber’s subject might have differed from that used in the simulation due to the lack of 3D shape information. The predicted linear fracture in the simulation was shorter than observed in the test and failed to capture the subsequent fracture pattern, which veered slightly towardthe lambdoidal suture at the posterior end.

### Two legal cases skull fracture analysis

**Fig. 8:**
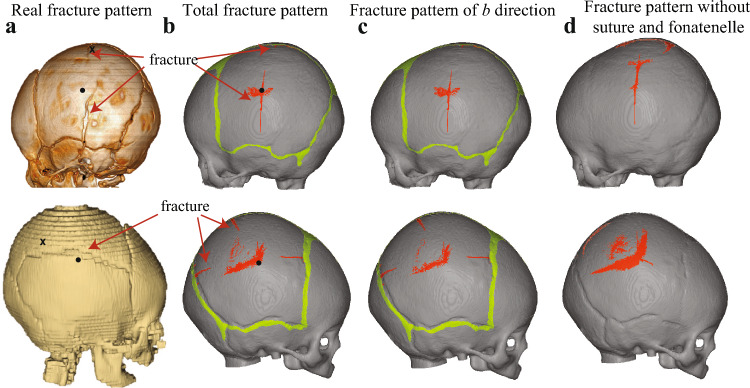
Fracture patterns are compared among CT scan from simulation (the upper part shows the 3 M case, and the lower part shows the 4 M case, locations of ossification centers are marked in black dots, and the impact locations are marked in black cross). **a** Fracture pattern in CT scans. **b** Total fracture pattern shown in simulation with anisotropic damage model. **c** Fracture pattern of *b* direction. **d** Total fracture pattern shown in simulation without any sutures or fontanelles (the material property was changed to be identical to the adjacent plate bones)

Results of applying different material properties (see Supplementary information for details) indicate that the predicted skull fractures are more conservative in cases with softer bone characteristics, while only a few damaged elements were observed in the other groups. Figure 8b,c shows the reconstructed skull fracture from two legal cases using the compliant bone material properties and compares the simulated overall fracture patterns, as well as those of the in-plane fiber perpendicular direction (*b*). Fracture patterns of the other two directions, combined with the evolution of the number of damaged elements during impact in different directions, are shown in supplementary discussion to investigate injuries occurring in different directions during infant skull fractures. The CT image from the 3 M case reveals two linear fractures in the left parietal bone. The longest fracture progresses along the fiber direction, extending from the point of swelling to the middle of the squamosal suture. In the simulation, this fracture initializes near the OC and propagate toward both the sagittal and squamosal sutures along the fiber direction but does not reach the cranial sutures. Another fracture seen in the CT scan is perpendicular to the sagittal suture and aligned with the fiber direction, which is accurately replicated in our simulation. For the 4 M case, the CT image reveals a fracture line parallel to the sagittal suture that spans the entire bone. Our simulation predicts three discontinuous fractures in the same area, along with a short fracture from sagittal suture toward to the OC. Both cases demonstrate that the majority of damage occur in directions perpendicular to the fiber, particularly in the in-plane direction, causing the fracture to propagate in the mineralization direction (fiber direction) from the ossification centers of the infant cranial plate bones. By altering the material properties of the sutures and fontanelles to be identical to those of the surrounding cranial plate bones, Fig. 8d demonstrates the skull fracture pattern in the absence of soft and compliant sutures and fontanelles. Apart from damage near the impact point, the primary fracture pattern still follows the grain fiber direction from the OC.

## Discussion

### Fractures mechanism

This study provides a previously lacking biomechanical explanation for the etiology of infant skull fractures, highlighting how intrinsic properties cause fractures that align with the direction of mineralization in the infant skull. It is not surprising that a majority of linear skull fractures either initiate or terminate at sutures (Kleinman et al. 2015). Our results indicate that the fractures primarily initiate from the sutures because of their softer and more compliant material properties compared to the adjacent cranial bones, which is consistent with the rule of skull fracture generation following blunt impact (Gunnarsson et al. 2021). Moreover, it has been reported that linear fractures often initiate from the microfractures generated near the suture (Gunnarsson et al. 2021) for adult skulls, and it is challenging for conventional CT examinations to detect such microfractures. In this study, the predicted fractures tend to align with the fiber direction, which is consistent with infant skull fractures seen clinically (Holck 2005; Ruiz-Maldonado et al. 2023). However, our results indicate that the weaker material properties of infant sutures are not the reason of infant skull fractures propagating along fibers. Instead, the softer sutures are prone to initiating fractures upon impact, which then extend toward the OC along the fibers.

**Fig. 9:**
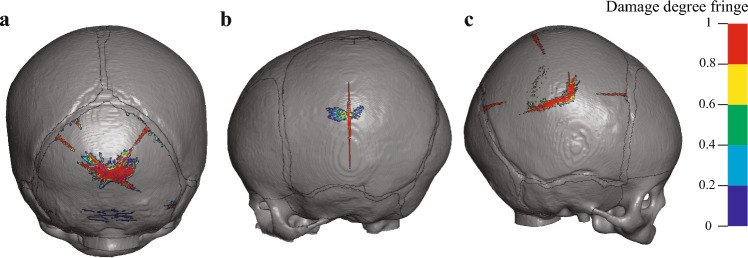
The damage degree contour plot in the skull. **a** Weber case A2. **b** Legal 3 M case. **c** Legal 4 M case

In the case of C5, the simulation results closely align with the Weber experiment, and all the fractures of A2 recorded in Weber’s experiment are also captured by simulation. However, the lack of further experimental details and the comprehensive skull characteristics increases the uncertainty of the results. For the two legal cases, the reconstructed fractures aligned well with the overall recorded results. Both cases indicated that multiple linear fractures could occur from a free fall of approximately 1 m. The results of the two reproduced legal cases also presented many nonlinear fracture patterns that are different from the CT images. This discrepancy arises from the characterization of fracture patterns in this paper, which is based on the identification of damaged elements, rather than considering variations in the extent of damage among different elements. In our study, the extent of damage is quantified by the scalar damage value $\varphi _j$, as shown in Eq. (21), where 0 indicates no damage, and 1 represents complete damage. Figure 9 illustrates the quantification of damage degree, indicating the dominant damage mode in the simulation remains linear, with areas of irregular flaky patterns exhibiting less severe damage compared to the primary linear fractures. The bone in these areas was partially damaged, but lacked sufficient energy for further microfracture propagation. In practice, the reconstruction of real fractures is very complicated due to the ductility of infant skulls, which needs more and deeper experimental verification in the future.

Lissner et al. (Lissner and Evans 1956) investigated various types of fractures and reported that linear fractures are more likely to occur with the minimum required impact energy than other types of fractures. It has been reported that for children 64.6 percent of skull fractures are linear, and the majority of these fractures occur near the suture (Lee et al. 2019). It is generally believed that linear skull fractures are the result of head impacting on the flat plane at a relatively low speed, while stellate fractures result from a high-velocity impact on the flat plane and depressed fractures are generated from the high-energy impact with a small contact area (DiMaio and DiMaio 2001). For infants, it is evident that the development of the infant skull follows the growth of fibers radiating from the ossification center. The fracture patterns of various directions observed in this study validate that impact-induced infant skull fractures are more prone to propagate along the mineralization direction, which also aligns with observations from cases of infant skull fractures (Holck 2005). For both cases, the in-plane direction perpendicular to the fiber is the major fracture direction and more representative of the overall fracture pattern.

### The importance of transversely isotropic material for simulating infant skull fracture

**Fig. 10:**
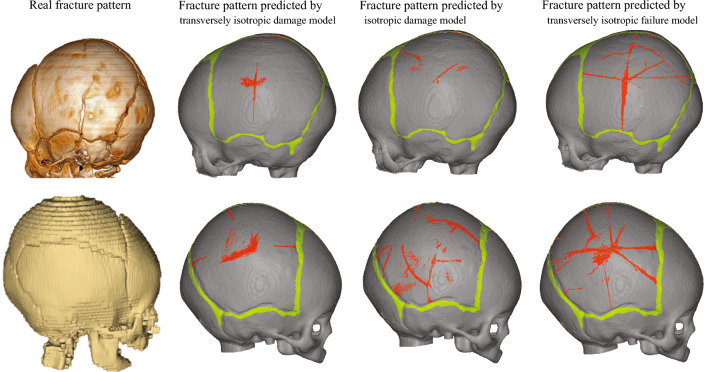
Comparison of fracture patterns in different material models (3 M legal case shown in the upper row, and 4 M legal case in the lower row). In the transversely isotropic material model, material properties along and perpendicular to the fiber direction were respectively fitted based on actual material tests (details in the Sect. 2.2.2). In the isotropic material model, both the material property and damage parameters along the fiber direction were adjusted to match those perpendicular to the fiber direction. Compared with the result by the transversely isotropic material model, the fracture pattern predicted by the isotropic material model differed significantly from the real fracture pattern observed in CT images. In the failure material model, the damage parameters were set to zero to eliminate the ductile properties

Figure 10 elucidates the pivotal role of transversely isotropic material properties in the reconstruction of infant skull fractures. In the isotropic material model group, the material and damage properties along the mineralization direction are aligned with those in the direction perpendicular to the mineralization, showing uniformity across both orientations. A comparative analysis between the transversely isotropic material model and the isotropic material model revealed a marked disparity in the resultant fracture patterns. Moreover, the fracture pattern predicted by the isotropic material model diverged significantly from the actual fracture pattern observed. Consequently, isotropic material models are inadequate in replicating the anisotropic material properties of the infant skull which have stiffer and stronger properties in the mineralization direction and weaker in the perpendicular direction. Although several fractures are still associated with the sutures, most of fracture lines do not align with the mineralization direction. Additionally, the differences between the failure model and the damage model are also illustrated in Fig. 10. Due to its inability to capture the ductile property of an infant’s skull, the failure model predicts a fracture pattern that is both more severe and complex than that predicted by the damage model. Even though the fracture patterns reconstructed by the damage model include the actual fractures observed in CT scans, it still overestimates the number of fracture lines that would not occur in reality. Therefore, it was essential to employ anisotropic damage material model that incorporates experimentally derived properties for both directions to reproduce the characteristic fracture lines along the mineralization directions observed in infant skull fractures.

### Comparison with infant head impact experiments

Experimental studies on human head impact are invaluable, especially when it comes to a pediatric head. Multiple experiments and simulations have demonstrated that the significant influence of impact location on head dynamics (Wright and Laing 2012; Jones et al. 2017; Loyd et al. 2019; Li et al. 2017). In general, both our simulation results and the experiment (Loyd et al. 2019; Prange et al. 2004) indicate that peak acceleration and corresponding impact stiffness increase, while impact duration decreases with escalating impact height. However, for the P12M forehead impact test and the P13F left parietal bone impact, it is noteworthy that the maximum acceleration at 15 cm exceeds that at 30 cm (Loyd et al. 2019), potentially attributable to sample reuse during the test. Besides that, Loyd (Loyd et al. 2019) reported the general trend that younger samples were more compliant than older samples with lower peak forces and longer impulse durations by comparing the different results between neonates, toddlers, and youth, which is identical to the trend from our simulation results, where an increase in the elastic modulus of infant skull corresponds to larger peak force and shorter impulse duration. On the other hand, the fluctuant force plateau in the force-time curve is considered as one of the potential indicators of skull fractures ( Loyd (2011), Chapter 6). Our results and the previous study (Li et al. 2017) indicate that the complex fluctuations in the force-time curve are more likely to be influenced by the location of the impact point and not directly related to the occurrence of fractures. Compared to other regions of the skull, impact on the parietal bones could result in greater fluctuations in the force-time curve.

### Limitations and future work

The numerical results reported herein should be considered in light of certain limitations. The primary limitation affecting the generalizability of these results comes from the lack of mechanical testing on infant skulls and other soft tissues of the infant head, which arises due to the scarcity of available pediatric cadaver donors and the ethical considerations surrounding their utilization. Firstly, in our infant skull material model, we have adapted several assumptions, such as assuming that the material properties of the frontal bones and the temporal bones are the same as those of the infant parietal skull in this study, as well as the estimation of the compressive and shear strength of the infant skull, which is due to the unavailability of relevant high-rate material property experiments for these material parameters. Secondly, the parameters of scalp material used in this paper are scaled using data collected from adults. In infants, the scalp is softer and thinner in comparison to adults, which may account for the simulated global acceleration curves exhibiting higher values than those observed in the actual test curve. Finally, since all the impact scenarios discussed in this paper involve hard flooring, all collision objects in the simulations are modeled as rigid flat plates. This study lacks discussion on different impact surfaces, such as carpets and foam flooring, for instance.

In general, infant skull fractures are associated with sutures, and there are anatomical differences between various infant sutures. The influence of the mechanical and geometric properties of sutures on infant skull fractures remains uncertain. Given that infant skull fractures are commonly associated with sutures, the authors believe that it is crucial to model the infant sutures in a detailed way and investigate their effects on infant skull fractures systematically. Furthermore, sensitivity studies on the material properties of various cranial bones are needed to better understand infant skull fractures, considering the uncertainty surrounding infant skull materials.

## Conclusion

In this study, the progressive damage unidirectional fabric composite model is combined with nonlinear soft tissue material models to investigate the impact response of the infant skull using CT-based subject-specific FE models. The methods of modeling the infant skull are verified by the three points bending simulation. Furthermore, these models are validated against the Loyd drop test (Loyd 2011) and the Weber fracture case reconstruction (Weber 1984).

This study reveals that the underlying reason for infant fractures extending along the mineralization direction is the significant anisotropy of the infant skull. The soft and compliant sutures of infants make fractures more likely to initiate near the sutures; however, the substantial material property variation between the sutures and infant bones are not the reason for fractures extending along the mineralization direction. We found that the peak force increased and the impact duration decreased as the elastic modulus of the infant skull increased. Moreover, the impact location and height significantly influence the fracture pattern and dynamic response. As the fall height increases, the maximum force increases, and the duration becomes shorter. Furthermore, our findings demonstrate that even among infants within the same age group, variations in cranial biomechanical properties result in distinct fracture patterns. Especially falls from approximately 1-meter in height can induce multiple linear fractures in the infant skull, which could be valuable information to aid the forensic investigations and the legal community in identifying abuse and accidents.

In summary, this study elucidated the inherent alignment of infant skull fractures with the direction of mineralization from the biomechanical perspective. The presented methods are capable of predicting the fracture patterns of infant skulls after drop. This advancement provides novel insights into the precise assessment of infant fractures, laying the groundwork for more objective biomechanics evidence in instances of alleged abuse, as well as facilitating biomechanical analyses of traumatic injuries in infants. This contributes to the field by offering a scientifically robust framework for understanding and investigating the biomechanics behind infant skull fractures.

## Supplementary Information

Below is the link to the electronic supplementary material.

Supplementary file 1 (pdf 2076 KB)

## Appendix A

The progressive damaged model used in this study has six failure criteria along the fiber direction (Eqs. 11, 14), the in-plane perpendicular to the fiber direction (Eqs. 12, 15), and the out-of-plane through the thickness direction (Eqs. 13, 16).

11 $$\begin{aligned} &  f_1-r_1^2=\left( \frac{\sigma _a}{ S_{a\textrm{T}}}\right) ^2 +\left( \frac{\tau _{ab}^2+\tau _{ac}^2}{ S_\textrm{FS}^2}\right) -r_1^2= 0 \end{aligned}$$

12 $$\begin{aligned} &  \quad f_2-r_2^2=\left( \frac{\sigma _b}{ S_{b\textrm{T}}}\right) ^2 +\left( \frac{\tau _{ab}}{ S_{ab}}\right) ^2+\left( \frac{\tau _{bc}}{ S_{bc}}\right) ^2-r_2^2= 0 \end{aligned}$$

13 $$\begin{aligned} &  \quad f_3-r_3^2=\left( \frac{\sigma _c}{ S_{c\textrm{T}}}\right) ^2 +\left( \frac{\tau _{ac}}{ S_{ac}}\right) ^2+\left( \frac{\tau _{bc}}{ S_{bc}}\right) ^2-r_3^2= 0 \end{aligned}$$

14 $$\begin{aligned} &  \quad f_4-r_4^2=\left( \frac{E_a\left\langle \varepsilon _a^\prime \right\rangle }{ S_{a\textrm{C}}}\right) ^2 -r_4^2= 0 \end{aligned}$$

15 $$\begin{aligned} &  \quad f_5-r_5^2=\left( \frac{E_b\left\langle \varepsilon _b^\prime \right\rangle }{ S_{b\textrm{C}}}\right) ^2 -r_5^2= 0 \end{aligned}$$

16 $$\begin{aligned} &  \quad f_6-r_6^2=\left( \frac{E_c\left\langle \varepsilon _c^\prime \right\rangle }{ S_{c\textrm{C}}}\right) ^2 -r_6^2= 0 \end{aligned}$$

The damage criteria $f_i-r_i^2=0$ of Eqs. (11–16) provide damage surfaces in the strain space, where $S_{i\textrm{T}}, S_{i\textrm{C}} (i=a,b,c)$ represent the tension and compressive strength along the direction *i*, $S_{ab}$, $S_{bc}$, $S_{ac}$ signifies the shear strengths, and $S_{\textrm{FS}}$ denotes the strength associated with fiber shear failure, $E_i (i=a,b,c)$ corresponds to the Young’s modulus along the *i* direction and $\langle \rangle$ is the Macaulay bracket. Additionally, $\varepsilon _a^\prime =-\varepsilon _a-\frac{-E_c\varepsilon _c-E_b\varepsilon _b}{2E_a}$ represents the adjusted strain, where $\varepsilon _i^\prime (i=b,c)$ denotes the compressive strain along the *i* direction. Meanwhile, $r_j (j = 1,...,6)$ indicates the damage factor with thresholds of 1 before damage initialization, which are updated as damage development progresses. Six damage variables $\omega _i$ with $i=1,...,6$ represent the initiation and progression of anisotropic damage (Matzenmiller et al. 1995). The compliance matrix [*S*], is the function of damage variables, where $\alpha _i=1-\omega _i (i=1,...,6)$ and the default term is zero.

$$ [S] = \left| {\begin{array}{*{20}c}    {\frac{1}{{\alpha _{1} E_{a} }}} & {\frac{{ - \mu _{{ba}} }}{{E_{b} }}} & {\frac{{ - \mu _{{ca}} }}{{E_{c} }}} & {} & {} & {}  \\    {\frac{{ - \mu _{{ba}} }}{{E_{b} }}} & {\frac{1}{{\alpha _{b} E_{b} }}} & {\frac{{ - \mu _{{cb}} }}{{E_{c} }}} & {} & {} & {}  \\    {\frac{{ - \mu _{{ca}} }}{{E_{c} }}} & {\frac{{ - \mu _{{cb}} }}{{E_{c} }}} & {\frac{1}{{\alpha _{c} E_{c} }}} & {} & {} & {}  \\    {} & {} & {} & {\frac{a}{{\alpha _{4} G_{{ab}} }}} & {} & {}  \\    {} & {} & {} & {} & {\frac{a}{{\alpha _{5} G_{{bc}} }}} & {}  \\    {} & {} & {} & {} & {} & {\frac{a}{{\alpha _{6} G_{{ca}} }}}  \\   \end{array} } \right| $$

The growth rate of damage variables, $\dot{\omega }_i$, is governed by the following damage rule:

17 $$\begin{aligned} \dot{\omega }_i=\textrm{max}\left\{ \dot{\varphi }_jq_{ij} \right\} (i,j=1,...,6) \end{aligned}$$

where *q* is the damage coupling matrix and Eq.18 illustrates how the term is associated with the modulus reduction, and $\dot{\varphi }_j$ is the scalar damage function to control the amount of damage growth.

18

$\dot{\varphi }_j$ is assumed to have the form:

19 $$\begin{aligned} \dot{\varphi }_j={\sum _{i}}{\gamma _j\frac{\partial f_j}{\partial \varepsilon _i}\dot{\varepsilon }_i} \end{aligned}$$

where the damage growth function is

20 $$\begin{aligned} \gamma _j= \frac{1}{2}(1-\varphi _j)f_j^{\frac{m_j}{2}-1} \end{aligned}$$

where $m_j$ is the damage parameter related to the strain softening behavior of the $j^\textrm{th}$ failure mode. By integrating Eq. (19), the scalar damage function $\varphi _j$ is expressed as follows:

21 $$\begin{aligned} \varphi _j=1-\textrm{exp}\left[ \frac{1}{m_j}\left( 1-r_j^{m_j}\right) \right] \end{aligned}$$

The damage threshold $r_j$ can be expressed as the ratio between the current total strain $\varepsilon _j$ and the corresponding yield strain $\varepsilon _{jy}$.

22 $$\begin{aligned} r_j=\frac{\varepsilon _j}{\varepsilon _{jy}} \end{aligned}$$

From Eq. (22), the Young’s modulus can be expressed as below.

23 $$\begin{aligned} E_j=(1-\omega _j)E_{j0}=E_{j0}\textrm{exp}\left[ \frac{1}{m_j}\left( 1-\left( \frac{\varepsilon _j}{\varepsilon _{jy}}\right) ^{m_j}\right) \right] \end{aligned}$$

Therefore, the stress of the damaged material can be expressed as

24 $$\begin{aligned} \sigma _j=E_j\varepsilon _j=E_{j0}\varepsilon _j\textrm{exp}\left[ \frac{1}{m_j}\left( 1-\left( \frac{\varepsilon _j}{\varepsilon _{jy}}\right) ^{m_j}\right) \right] \end{aligned}$$
