# Supplementary material for: Infant skull fractures align with the direction of bone mineralization
Source: Biomech Model Mechanobiol. 2024 Nov 25;24(1):153–68. doi: 10.1007/s10237-024-01902-x (PMC11846741; doi:10.1007/s10237-024-01902-x)
Supplement: Supplementary file 1 — (pdf 2076 KB) [file 10237_2024_1902_MOESM1_ESM.pdf]

# 1 Supplementary discussion about infant skull fractures

Figure.1 (a) and (b) show the reconstructed skull fracture of the two legal cases with different material properties. The results of applying different material properties indicate that the predicted skull fractures are more conservative in cases with softer bone characteristics, while only a few damaged elements were observed in the other groups. Several radiology studies have shown that the capability of current CT technology in measuring bone thickness is limited, which may lead to significant overestimation (Treece et al. 2010; Museyko et al. 2017). Furthermore, most current finite element head models are largely developed based on CT images. The resolutions of the CT images for the 3M case and 4M case are $0.32 \times 0.32 \times 0.63\,\mathrm{mm}^3$ and $0.32 \times 0.32 \times 1.5\,\mathrm{mm}^3$, respectively. Limited by the accuracy of the employed CT technology, the current head finite element models might be thicker in the thickness direction than they actually are, such as the thicknesses of the CSF, dura mater, and cranial bone. The softer and more compliant skull material properties mitigate the increased actual stiffness caused by overestimation. Therefore, in these two cases, the simulation results using the base material parameters in these two cases do not exhibit a similar linear fracture pattern, whereas the predicted fracture patterns in the compliant material group are close to the actual CT images of skull fractures.

To investigate injuries occurring in different directions during infant skull fractures, Figure.2 compares the simulated overall fracture patterns as well as in different directions. Figure.3 illustrates the evolution of the number of damaged elements combined with the fracture pattern in different directions. In the case of 3M scenario, the contact force reached its peak 5 milliseconds after the collision, yet the curve for the damaged element did not converge at this time, suggesting that the fracture formed subsequent to the peak of contact force. The force-time curve of 4M case exhibited multiple fluctuations. Similarly, the final fracture pattern was not fully formed when the force reached its maximum. The curve representing the number of damaged elements converges at the endpoint of the force-time curve's fluctuations. All cases demonstrate that minimal damage along the fiber during infant skull fractures, with the majority of damage occurring perpendicular to the fiber, particularly along the direction of in-plane. Besides that, damage pattern along the in-plane perpendicular direction is most close to the overall damage pattern, which means infant skull fractures are mainly induced by damage perpendicular to the trabecular fibers.

# References

Oleg Museyko, Bastian Gerner, and Klaus Engelke. A new method to determine cortical bone thickness in ct images using a hybrid approach of parametric profile representation and local adaptive thresholds: Accuracy results. *PLoS One*, 12(11):e0187097, 2017.

Graham M Treece, Andrew H Gee, PM Mayhew, and Kenneth ES Poole. High resolution cortical bone thickness measurement from clinical ct data. *Medical image analysis*, 14(3):276–290, 2010.

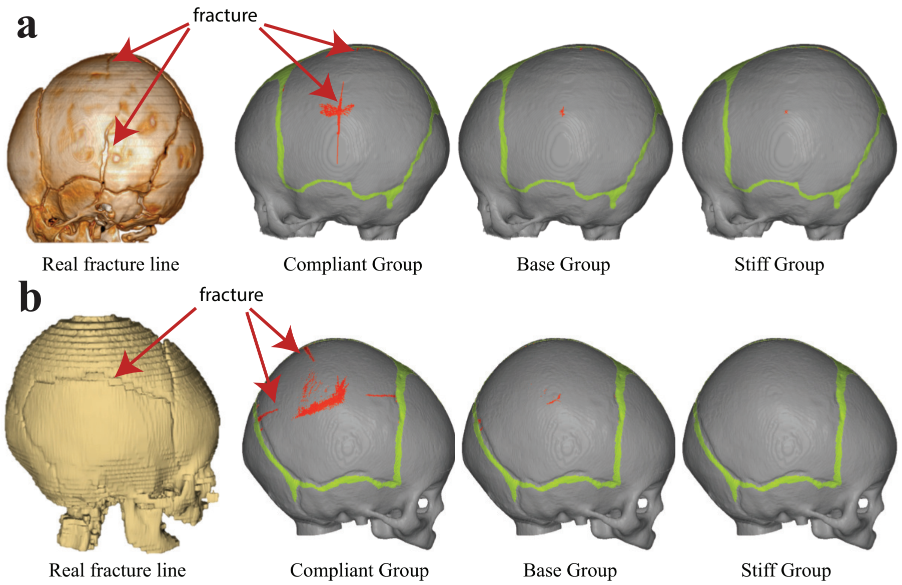


Figure 1: **Two legal cases skull fracture analysis a,** The skull fractures shown from the CT and the reconstructed fracture pattern of three different material properties of 3M case, where the compliant group uses the 95% CI lower elastic modulus boundary in conjunction with the 95% CI lower strength values, the base group uses the fitted value of elastic modulus and strength values, the stiff group uses the 95% CI upper boundary in conjunction with the 95% CI upper strength values. **b,** The skull fractures shown from the CT and the reconstructed fracture pattern of three different material properties of 4M case.

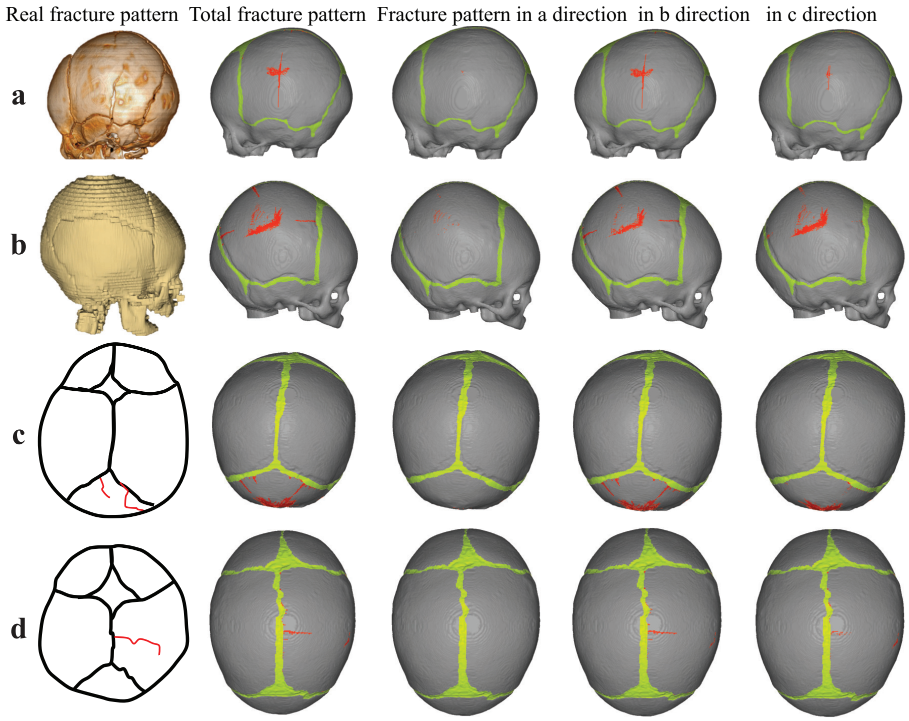


Figure 2: **Fracture patterns in different directions, where the first column shows the real fracture pattern, the second column shows total damage pattern by simulation, and the last three columns show the damage pattern in a, b, and c directions respectively.** **a,** The reconstructed legal case of 3M. **b,** The reconstructed legal case of 4M. **c,** Weber case A2. **d,** Weber case C5.

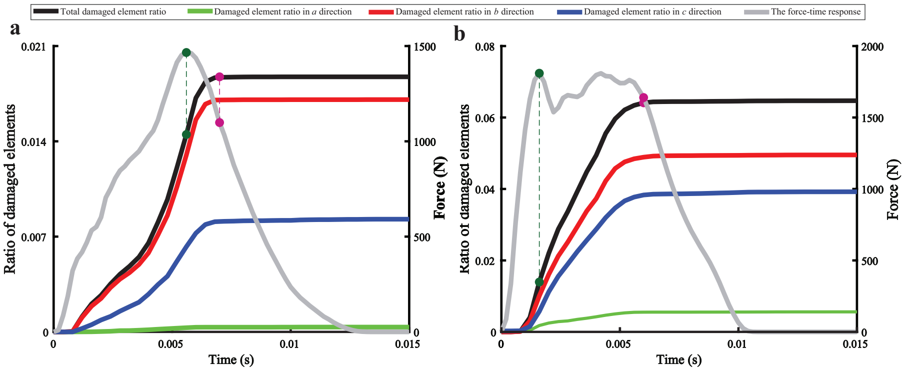


Figure 3: **a,** The ratio of damage elements in three direction of the compliant group of 3M case and the corresponding force-time curve. The darkgreen dot indicates the moment when the contact force reaches its peak and the dot in purple is the convergence point of the total number of the damage element. **b,** The ratio of damage elements in three direction of 4M case and the corresponding force-time curve.
